# Links Between Staffing and Resource Inadequacy and Missed Nursing Care in an Academic Medical Center (Eastern Province, Saudi Arabia): A Cross-Sectional Study

**DOI:** 10.3390/nursrep16020069

**Published:** 2026-02-15

**Authors:** Ayat Ali Al-Sawad, Heba Adnan Dardas, Laila Hussain Al-Shawaf, Moudi Ayadah Shammari, Rabab Salman Emshamea, Ezdehar A. Al-Barbari, Mohammed Al-Hariri

**Affiliations:** 1 Department of Nursing Services, King Fahad University Hospital, Imam Abdulrahman Bin Faisal University, Alkhobar 34445, Saudi Arabia; 2 Department of Physiology, College of Medicine, Imam Abdulrahman Bin Faisal University, Dammam 31441, Saudi Arabia

**Keywords:** nurse, workload, health resources, patient safety, cross-sectional studies, Saudi Arabia

## Abstract

**Background:** Missed nursing care, defined as essential patient care that is omitted or delayed, is a growing source of concern due to its effects on healthcare quality and patient safety. Our aims in this study were twofold: first, we examined the extent and types of missed nursing care, and second, we analyzed the relationship between the care missed by hospital nurses and the staffing and resource adequacy in an academic medical center. **Methods:** A descriptive cross-sectional study was conducted during the period between November 2022 and July 2023. Data were collected using a self-administered questionnaire that comprised items on socio-demographic and work-related characteristics, items on staffing and resource availability, and items from the ‘MISSCARE’ Survey. **Results:** The most frequently missed nursing care involved pressure-relieving interventions (Mean = 2.39) and ambulation/mobilization (Mean = 2.27), while medication administration (Mean = 1.60) and glucose monitoring (Mean = 1.56) were missed the least. Labor resource inadequacy (β = 0.315, *p* < 0.001) and communication and teamwork deficits (β = 0.285, *p* < 0.001) were positively associated with missed nursing care, whereas staffing and resource adequacy showed an inverse association (β = −0.164, *p* = 0.006). The model explained 49.8% of the variance in missed nursing care (R^2^ = 0.498). **Conclusions:** These findings highlight that missed nursing care is a system-level issue primarily associated with staffing and resource constraints rather than individual characteristics. Improving staffing adequacy, resource availability, and interprofessional collaboration may reduce care omissions and enhance patient safety in Saudi Arabian academic medical centers.

## 1. Introduction

A nurse is often the first point of contact in the healthcare system, acting as a planner, coordinator, provider, and evaluator. Resource constraints and multifunctional demands force nurses to make difficult decisions about the quality of care they provide; it is common for nurses to be unable to meet standards of care due to various adverse circumstances. As a result of these critical situations, nurses may shorten, delay, or exclude the usual duration of care [1]. Missed nursing care refers to any form of required patient care that is either partially or completely omitted or significantly delayed; it is recognized as a serious threat to patient safety by international quality and safety standards [2]. This phenomenon has also been described as unfinished care, unmet nursing care needs, or implicit rationing of care [3].

A growing body of evidence indicates that missed nursing care is a critical indicator of healthcare quality and is consistently associated with adverse patient outcomes, including infections, medication errors, falls, and reduced patient satisfaction [4,5].

At the organizational level, missed nursing care reflects systemic challenges such as inadequate staffing, insufficient material resources, and suboptimal communication and teamwork. Numerous studies demonstrate that adequate staffing and resource availability are associated with improved patient outcomes, whereas staffing shortages and poor resource adequacy significantly increase the likelihood of essential care activities being omitted [6,7].

Globally, studies have shown that staffing shortages and limited resources significantly increase the likelihood of nurses omitting essential care activities [8,9]. Furthermore, a study conducted in university hospitals found that poor staffing and inadequate resources were significantly associated with higher rates of missed nursing care, which in turn correlated with lower perceived quality of care and increased adverse events [6].

Despite this robust collection of international literature, context-specific evidence from Saudi Arabia remains limited. Although some studies have begun to explore missed nursing care practices and their contributing factors within Saudi healthcare settings, such research is relatively recent and sparse. For instance, a cross-sectional study conducted in Jeddah identified that inadequate labor and material resources, along with issues with teamwork, were prevalent reasons for missed nursing care among registered nurses [10]. Additionally, broader studies on the characteristics of Saudi hospital nursing units have noted associations between safety culture, staffing perceptions, and nursing outcomes, suggesting an indirect relationship with care omissions that warrants further investigation [11].

Academic medical centers are particularly complex environments because of their high patient acuity, diverse workforce composition, and concurrent educational responsibilities. However, empirical research examining staffing and resource adequacy in relation to missed nursing care within Saudi academic medical centers is scarce.

Collectively, we aim to both fill a regional evidence gap and enrich international discourse by offering data from an underrepresented healthcare context, strengthening cross-cultural comparisons, and supporting the development of globally informed, locally responsive strategies to reduce missed nursing care. Our findings provide context-specific evidence to inform Saudi healthcare policy, hospital administrators, and nursing leaders about the modifiable organizational factors associated with missed nursing care. This aligns directly with the Saudi Vision 2030 priorities on healthcare quality, patient safety, workforce optimization, and evidence-based management. We aimed to both assess the extent and types of missed nursing care and to examine their associations with socio-demographic characteristics, unit-related factors, and perceived staffing and resource adequacy among nurses working in Saudi academic medical centers.

## 2. Materials and Methods

### 2.1. Study Design and Setting

A descriptive cross-sectional design was employed during the period between November 2022 and July 2023. The study population consisted of all staff nurses working at KFHU who provided direct patient care.

### 2.2. Sampling Technique

Participants were recruited using a convenience sample, with voluntary responses obtained using an open electronic survey distributed to all eligible nurses via Google Forms (Google LLC, Mountain View, CA, USA).

### 2.3. Inclusion and Exclusion Criteria

The inclusion criteria classified inpatient units as acute care units with generally short average lengths of stay (fewer than two days at the unit level), reflecting their high-turnover environment; this criterion was applied to the unit type rather than the duration that an individual patient was hospitalized for. Intensive Care Units (ICUs) were retained because they are administratively categorized as acute inpatient units and share core workflow characteristics with other high-acuity inpatient wards, including continuous bedside care, rapid clinical decision-making, and high task density. The following units were excluded from the study: short-stay units (23 h), operative units, dialysis, endoscopy, day surgery, birth rooms, outpatient clinics, and the emergency department. These areas operate under highly specialized, procedure-driven, or non-routine models of care that differ substantially from general inpatient ward environments. In particular, emergency departments are characterized by unpredictable patient flow, rapid turnover, and time-critical interventions.

### 2.4. Sample Size Calculation

The Raosoft sample size calculator (Raosoft Inc., Seattle, WA, USA; available online at https://www.raosoft.com/samplesize.html) was used to estimate the required sample size. Approximately 280 staff nurses were working in the eligible wards, of whom 98% were non-Saudi and 2% were Saudi. The confidence level was set at 95%, with a 5% margin of error, resulting in a minimum required sample size of 163 participants.

### 2.5. Recruitment Procedures

The study was introduced through electronic recruitment posters distributed to the nursing units. A secure link to the online questionnaire was sent to all registered nurses through the hospital’s official email system on behalf of the research team.

Before obtaining consent, the research team explained the study’s objectives, methodology, potential benefits, and safety assurances. Nurses were provided with the opportunity to ask questions before signing the consent form. They were informed of their right to withdraw at any time without any impact on their employment status.

### 2.6. Confidentiality and Participant Safety

The participating nurses received no compensation, and the study involved no known risks or adverse effects. All collected information was kept confidential and securely stored, and it was used exclusively for research purposes.

### 2.7. Data Collection Instruments

Tool I: Socio-Demographic and Unit-Related Characteristics:

This tool was used to collect information on participants’ age, gender, educational level, professional role, years of work experience, weekly work hours, overtime hours, and any missed shifts within the previous three months.

Tool II: Staffing and Resource Adequacy Subscale:

Staffing and resource adequacy were measured using the four-item subscale of the Practice Environment Scale of the Nursing Work Index (PES-NWI). Items were rated on a 4-point Likert scale ranging from 1 (strongly disagree) to 4 (strongly agree) [12].

Tool III: MISSCARE Survey:

The MISSCARE Survey, originally developed and later updated by Kalisch et al. (2009; 2019), was utilized to assess missed nursing care and its contributing factors [13].

Part A included 18 nursing care activities, with responses recorded on a 5-point Likert scale (1 = never missed, 2 = rarely missed, 3 = occasionally missed, 4 = frequently missed, 5 = always missed).

Part B included 19 items assessing the reasons for nursing care being missed, rated on a 4-point Likert scale (1 = not a reason, 2 = minor reason, 3 = moderate reason, 4 = significant reason).

We acknowledge that there is a conceptual relatedness between staffing and resource adequacy, labor resources, and material resources. However, staffing and resource adequacy refers to a higher-order evaluative judgment regarding whether overall staffing and resources are sufficient to meet care demands, whereas labor resources and material resources reflect perceptions of the availability of specific human and physical inputs. This distinction is consistent with prior frameworks of the nursing work environment, in which global assessments of staffing adequacy are treated as conceptually separate from indicators of specific resource availability (e.g., the PES-NWI and MISSCARE frameworks). Accordingly, these constructs were modeled as distinct but related dimensions, in line with the original instrument structure. This modeling decision is further supported by satisfactory discriminant validity based on HTMT criteria, indicating that the constructs capture related yet non-redundant aspects of the work environment.

### 2.8. Statistical Analysis

Data were analyzed using the Statistical Package for Social Sciences (SPSS) version 25.0. For categorical variables, descriptive statistics were presented as number (n) and percentage (%), and for continuous variables, they were presented as means (95% CI). Partial Least Squares Structural Equation Modeling (PLS-SEM) was employed to test the proposed research model due to its suitability for complex models and its robustness when applied to relatively small sample sizes. All analyses were conducted using SmartPLS software, version 4.0.9; Smar, Germany. Common method variance (CMV) was assessed using Harman’s single-factor test as a post hoc diagnostic procedure. The first unrotated factor accounted for 40.62% of the total variance, which is below the recommended threshold of 50%, suggesting that common method bias was unlikely to be a serious concern in this study.

The measurement model was evaluated by examining internal consistency reliability, convergent validity, and discriminant validity. Internal consistency reliability was assessed using Cronbach’s alpha and composite reliability (CR). Convergent validity was evaluated using indicator loadings and average variance extracted (AVE), while discriminant validity was examined using the Fornell–Larcker criterion and the heterotrait–monotrait (HTMT) ratio. The structural model was assessed by estimating path coefficients, coefficient of determination (R^2^), effect sizes (f^2^), and predictive relevance (Q^2^) using a bootstrapping procedure with 5000 resamples.

## 3. Results

### 3.1. Demographic Characteristics

Of the 280 nurses who received the survey link, 181 completed the questionnaire, yielding a response rate of 64.4%. Only fully completed surveys were included in the final analysis.

The sample was predominantly composed of middle-aged, female nurses with bachelor’s degree-level education. Most participants worked in adult surgical units and intensive care settings and reported substantial clinical experience, including long tenure in their current units. The majority worked extended hours and 12 h shifts, with many rotating between shifts, while a considerable proportion reported no overtime and no missed workdays. Overall, these findings reflect an experienced and highly committed nursing workforce (Table 1).

### 3.2. Descriptive Statistics

#### Missed Nursing Care

Activities related to patient mobility, pressure injury prevention, interdisciplinary coordination, and timely nutrition were identified as the most commonly omitted aspects of nursing care, suggesting that these domains are particularly susceptible to omissions in the studied setting (Table 2). Conversely, core clinical tasks such as medication administration and glucose monitoring were reported as least frequently missed, indicating relatively high compliance with these fundamental safety-related practices.

With respect to the perceived reasons for missed nursing care, the participants ranked limitations in labor and material resources most highly, underscoring this field’s status as a prominent organizational concern, followed by challenges related to communication and teamwork; staffing and resource adequacy was the lowest-ranked area (Table 3). Furthermore, discriminant validity assessed using the heterotrait–monotrait (HTMT) ratio demonstrated adequate distinction between the latent constructs (Table 4).

**Figure 1:**
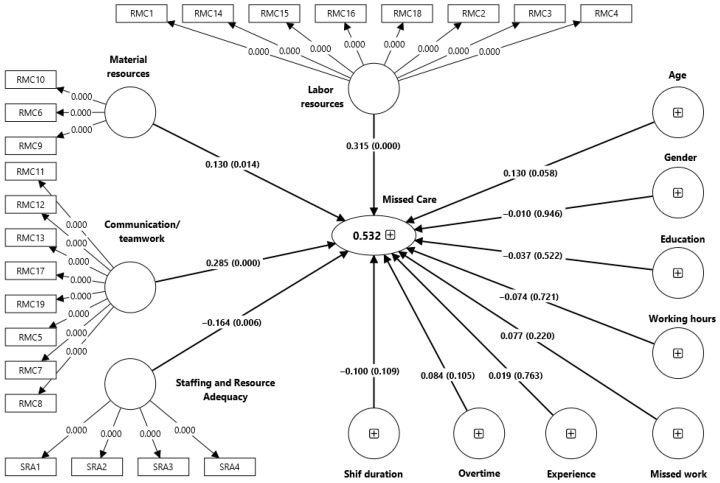
Bootstrapped structural model.

The structural model was evaluated after confirming the validity and reliability of the measurement model. PLS-SEM was utilized to test the hypothesized relationships between communication/teamwork, labor resources, material resources, staffing and resource adequacy (independent variables), and total missed nursing care score (dependent variable) (Figure 1).

Labor resources, communication and teamwork, and material resources were all significantly associated with missed nursing care, with labor resources and communication/teamwork showing the strongest relationships. In contrast, staffing and resource adequacy demonstrated a protective association, indicating that better staffing and resource availability are linked to reduced incidences of missed nursing care. Demographic and work-related characteristics were not significantly related to missed nursing care. Collectively, the structural model accounted for nearly half of the variance in missed nursing care and showed adequate predictive relevance (Table 5 and Table 6).

## 4. Discussion

The findings of the current study reveal distinct patterns in the nursing care activities that are most frequently omitted and underscore the relevance of organizational factors, particularly labor resources, communication and teamwork, and overall staffing and resource adequacy, as key correlates.

Consistent with prior international research, ambulation and pressure-relieving interventions were reported to be the most frequently missed nursing care activities. These tasks, which require sustained time, adequate staffing, and coordinated patient handling, have been identified as highly susceptible to omission in contexts characterized by high workload and resource constraints. For example, studies in acute care settings in the United States and Europe similarly report that mobility and repositioning activities are commonly omitted due to competing demands and limited time availability [14,15].

In this study, interdisciplinary care conference attendance and feeding patients while food was at the proper temperature were also frequently reported as missed. These observations are consistent with previous evidence from the Saudi Arabian healthcare context [16]. Reduced participation in interdisciplinary meetings may be associated with challenges in shared decision-making and case coordination, which are essential for managing complex patient conditions. Similarly, delayed or rushed feeding practices may be associated with risks to nutritional adequacy and recovery, particularly among older adults and patients with impaired oral intake.

Conversely, timely medication administration and ordered glucose monitoring were among the least frequently missed activities, reflecting strong adherence to core procedural standards. This pattern may relate to the presence of established protocols, electronic alert systems, and the clear linkage of these tasks to immediate patient safety outcomes. The prior literature suggests that when resources are constrained, nurses tend to prioritize tasks perceived as life-critical or audit-sensitive, such as medication delivery and physiological monitoring, over more time-intensive or discretionary activities [17]. This prioritization supports the conceptualization of missed nursing care as an indicator of care rationing under conditions of limited capacity rather than as individual negligence.

Our structural model highlights the role of organizational determinants in cases of missed nursing care. Labor resources had a significant positive association, indicating that perceptions of staffing shortages are linked to higher reported rates of care omissions. This finding is consistent with a systematic review demonstrating strong associations between nurse staffing levels and care quality outcomes, whereby lower staffing levels are associated with increased omissions, delays, and adverse events [18].

Communication and teamwork also exhibited a significant positive relationship with missed nursing care, underscoring the importance of collaborative practice environments in shaping care processes. Deficiencies in communication have been widely documented to be associated with care omissions, medical errors, and fragmented service delivery [19].

Although material resources showed a smaller effect size than labor resources or teamwork, a significant association was still present, indicating that limitations in physical and organizational resources are relevant to missed nursing care. Shortages in essential supplies, equipment, or supportive infrastructure may constrain nurses’ ability to complete required tasks efficiently, particularly those that are resource-intensive. In contrast, staffing and resource adequacy demonstrated a negative association with missed nursing care, suggesting that more favorable staffing environments and resource availability are associated with lower levels of reported omissions. This pattern is consistent with evidence describing the buffering role of supportive practice environments in relation to missed nursing care and patient outcomes [4].

Interestingly, demographic and work-related variables did not demonstrate significant effects, suggesting that missed nursing care is more strongly influenced by structural- and unit-level conditions than by individual characteristics, supporting related theoretical perspectives [20].

Collectively, the model accounted for almost half of the variance in missed nursing care, indicating that the included constructs provide meaningful explanatory power. The predictive relevance confirmed through Q^2^ further supports the robustness of these associations within the context of academic medical centers. Our discriminant validity assessments confirmed the distinctiveness of the latent constructs, increasing confidence in the validity of our measurement model.

The observed associations between labor and material resources, staffing and resource adequacy, and missed nursing care indicate that efforts to address missed nursing care may benefit from approaches that extend beyond staffing levels and incorporate broader organizational- and system-level considerations. From the perspective of practice, these findings imply the potential value of acuity-based staffing approaches, routine monitoring of workload distribution, and ensuring the continuous availability of essential supplies. Similarly, initiatives that promote effective communication and teamwork—such as interdisciplinary rounds, structured handover processes, and team-training programs—may be relevant in contexts where coordination-related omissions are prominent [13].

At the policy level, our results support the development of standards that incorporate patient acuity, skill mix, and unit complexity in workforce planning rather than relying on fixed nurse-to-patient ratios alone. Incorporating missed nursing care indicators into quality and patient safety monitoring systems may also offer useful insights into system performance, alongside investments in digital staffing platforms and real-time resource management tools [21].

Within academic medical centers, our findings further imply the importance of acknowledging the additional educational and preceptorship responsibilities of nurses when allocating time and resources. These considerations are consistent with the Saudi Vision 2030 healthcare transformation agenda, which emphasizes improving quality, patient safety, workforce optimization, and evidence-based management. Collectively, these implications highlight organizational domains that may warrant attention in efforts to better understand and address missed nursing care. However, given the cross-sectional nature of our data, potential endogeneity and reverse causality cannot be fully excluded. Therefore, the observed associations should not be interpreted as causal, and future longitudinal or multi-source studies are recommended to confirm causality.

### Limitations

This study has several limitations. Its cross-sectional design precludes the establishment of causal relationships or temporal sequencing between staffing, resource adequacy, and missed nursing care; therefore, the observed relationships should be interpreted as associative rather than causal, and reverse causation and residual confounding cannot be excluded. Data were collected using self-reported measures, which may be influenced by recall and social desirability bias and could lead to underestimation of care omissions; nevertheless, such methods remain important for capturing aspects of nursing care that are not routinely documented. Although validated instruments were employed, these tools were originally developed in Western healthcare contexts, and their applicability across different cultural and organizational settings may be affected by contextual differences despite appropriate translation and validation procedures. In addition, this study was conducted in a single academic medical center, which may restrict its generalizability to other healthcare settings with different organizational structures, staffing models, or levels of resource availability.

## 5. Conclusions

This study indicates that instances of missed nursing care in an academic medical center are more strongly associated with staffing and resource adequacy than with individual characteristics. Care activities requiring sustained time and coordination—particularly patient mobilization, pressure-relieving interventions, and interdisciplinary communication—were more frequently reported as missed, whereas medication administration and glucose monitoring were consistently prioritized. The observed associations between labor resources, teamwork, and missed nursing care highlight the importance of unit-level and organizational conditions in shaping patterns of care delivery. These results provide important implications for healthcare leaders and policymakers regarding the potential value of strengthening staffing models, optimizing resource availability, and supporting effective team communication as part of broader efforts to address missed nursing care and enhance patient safety and quality of care.

## Figures and Tables

**Table 1:** Frequency analysis of socio-demographic and work-related characteristics.

Variable	Level	Frequency	Percent
Age	<25	4	2.2
	25–34	72	39.8
	35–44	72	39.8
	45–54	31	17.1
	>55	2	1.1
Gender	Female	159	87.8
	Male	22	12.2
Education	Diploma	27	14.9
	Bachelor	152	84
	Master	2	1.1
The unit you work in	Adult Medical Unit	48	26.5
	Adult Surgical Unit	62	34.2
	ICU	49	27.1
	Pediatric Surgical Unit	2	1.1
	Pediatric Medical Unit	11	6.1
	Psychiatric Unit	9	5
Overtime	None	73	40.3
	1–12 h of overtime	62	34.3
	>12 h of overtime	46	25.4
Missed work	None	93	51.4
	1 day or shift	33	18.2
	2–3 days or shifts	40	22.1
	4–6 days or shifts	5	2.8
	Over 6 days or shifts	10	5.5
Experience in your role	Up to 6 months	6	3.3
	6+ months to 2 years	5	2.8
	2+ years to 5 years	27	14.9
	5+ years to 10 years	52	28.7
	Greater than 10 years	91	50.3
Experience in your current unit	Up to 6 months	15	8.3
	6+ months to 2 years	11	6.1
	2+ years to 5 years	48	26.5
	5+ years to 10 years	51	28.2
	Greater than 10 years	56	30.9
Working hours	Less than 48 h per week	19	10.5
	48 h or more per week	162	89.5
Duration of each shift	8 h	49	27.1
	10 h	21	11.6
	12 h	111	61.3
Time of shift	Day	54	29.8
	Evening	4	2.2
	Night	39	21.6
	Rotating between days, evening, and nights	84	46.4

ICU: Intensive Care Unit.

**Table 2:** Frequency analysis of missed nursing care indicators.

Item	Not Applicable	Never Missed	Rarely Missed	Occasionally Missed	Frequently Missed	Always Missed	Mean
Ambulation/mobilization	27(14.9)	30(16.6)	36(19.9)	49(27.1)	33(18.2)	6(3.3)	2.27
Pressure-relieving interventions	13(7.2)	33(18.2)	49(27.1)	50(27.6)	28(15.5)	8(4.4)	2.39
Feeding patients while food is still at the proper temperature	15(8.3)	55(30.4)	50(27.6)	31(17.1)	23(12.7)	7(3.9)	2.07
Setting up meals for patients who can feed themselves	17(9.4)	73(40.3)	44(24.3)	27(14.9)	14(7.7)	6(3.3)	1.81
Administering medications as scheduled	7(3.9)	99(54.7)	49(27.1)	14(7.7)	9(5)	3(1.7)	1.6
Assessment of vital signs	5(2.8)	105(58)	43(23.8)	15(8.3)	10(5.5)	3(1.7)	1.61
Monitoring intake/output	5(2.8)	98(54.1)	52(28.7)	16(8.8)	8(4.4)	2(1.1)	1.61
Full documentation of all care provided	3(1.7)	74(40.9)	56(30.9)	31(17.1)	14(7.7)	3(1.7)	1.93
Bathing/showering	16(8.8)	81(44.8)	45(24.9)	20(11)	16(8.8)	3(1.7)	1.71
Oral care	12(6.6)	75(41.4)	47(26)	26(14.4)	18(9.9)	3(1.7)	1.85
Glucose monitoring as ordered	4(2.2)	106(58.6)	49(27.1)	12(6.6)	8(4.4)	2(1.1)	1.56
IV/central line site care and assessments according to facility policy	7(3.9)	96(53)	45(24.9)	22(12.2)	9(5)	2(1.1)	1.65
Responding to call lights within 5 min	23(12.7)	61(33.7)	43(23.8)	29(16)	21(11.6)	4(2.2)	1.87
Acting on PRN medication requests within 15 min	11(6.1)	87(48.1)	47(26)	21(11.6)	13(7.2)	2(1.1)	1.69
Attending interdisciplinary care conferences when held	13(7.2)	54(29.8)	41(22.7)	29(16)	28(15.5)	16(8.8)	2.29
Assisting with toileting needs within 5 min of request	10(5.5)	68(37.6)	47(26)	32(17.7)	20(11)	4(2.2)	1.98
Skin/wound care	8(4.4)	82(45.3)	48(26.5)	25(13.8)	15(8.3)	3(1.7)	1.81
Adequate surveillance of confused/impaired residents	14(7.7)	75(41.4)	48(26.5)	26(14.4)	16(8.8)	2(1.1)	1.78

IV: intravenous; PRN: pro re nata.

**Table 3:** Descriptive statistics: constructs on reasons for missed nursing care and resource adequacy.

Variable	Scale	Min	Max	Mean	SD
Communication/teamwork	1 to 4	1	4	2.69	0.77
Labor resources	1 to 4	1.13	4	3.21	0.65
Material resources	1 to 4	1	4	3.08	0.89
Staffing and resource adequacy	1 to4	1	4	1.99	0.80

**Table 4:** Discriminant validity based on heterotrait–monotrait ratio.

	C/TW	LR	MC.t	MR
Communication/teamwork				
Labor resources	0.535			
Total missed nursing care score	0.565	0.638		
Material resources	0.507	0.591	0.526	
Staffing and resource adequacy	0.376	0.538	0.479	0.376

C/TW: communication/teamwork; LR: labor resources; MC.t: total missed nursing care score; MR: material resources.

**Table 5:** Bootstrapped path assessment model.

Path	β	SE	T Value	*p* Value	*f*^2^ Effect Size
Main factors					
Communication/teamwork -> Missed nursing care	0.285	0.054	5.236	<0.001	0.115
Labor resources -> Missed nursing care	0.315	0.061	5.15	<0.001	0.118
Material resources -> Missed nursing care	0.130	0.053	2.461	0.014	0.023
Staffing and resource adequacy -> Missed nursing care	−0.164	0.06	2.727	0.006	0.04
Demographic factors					
Age -> Missed nursing care	0.13	0.069	1.894	0.058	0.021
Education -> Missed nursing care	−0.037	0.057	0.64	0.522	0.002
Gender -> Missed nursing care	−0.010	0.151	0.068	0.946	0.001
Work-related factors					
Experience -> Missed nursing care	0.019	0.064	0.302	0.763	0.001
Missed work -> Missed nursing care	0.077	0.063	1.226	0.22	0.011
Overtime -> Missed nursing care	0.084	0.052	1.623	0.105	0.013
Shift duration -> Missed nursing care	−0.100	0.062	1.605	0.109	0.019
Working hours -> Missed nursing care	−0.074	0.207	0.358	0.721	0.001

**Table 6:** Structural model: quality indices of endogenous construct.

Endogenous Construct	R^2^	Q^2^
Missed nursing care	0.498	0.444

## Data Availability

The raw data supporting the conclusions of this article will be made available by the authors on request.

## Public Involvement Statement

No public involvement in any aspect of this research.

## Guidelines and Standards Statement

This manuscript was drafted against the Strengthening the Reporting of Observational Studies in Epidemiology (STROBE) guidelines for cross-sectional studies.
